# Characterization of milk oligosaccharide and sialic acid content and their influence on brain sialic acid in a lean mouse model for gestational diabetes

**DOI:** 10.1016/j.heliyon.2024.e24539

**Published:** 2024-01-24

**Authors:** Fan Liu, Angela J.C. Tol, Folkert Kuipers, Maaike H. Oosterveer, Eline M. van der Beek, Sander S. van Leeuwen

**Affiliations:** a Laboratory of Pediatrics, University of Groningen, University Medical Center Groningen, Groningen, the Netherlands; b European Research Institute for the Biology of Ageing (ERIBA), University of Groningen, University Medical Center Groningen, Hanzeplein 1, 9713 GZ, Groningen, the Netherlands; c Department of Laboratory Medicine, University of Groningen, University Medical Center Groningen, Groningen, Hanzeplein 1, 9713 GZ, Groningen, the Netherlands

**Keywords:** Mouse milk, Oligosaccharides, Sialic acids (sia), Brain, Gestational diabetes mellitus (GDM), Pre-gestational diabetes (PDM)

## Abstract

Oligosaccharides and sialic acids (Sia) are bioactive components in milk that contribute to newborn development and health. Hyperglycemia in pregnancy (HIP) can have adverse effects on both mother and infant. HIP is associated with low-grade systemic inflammation. Inflammation influenced glycan composition, particularly of Sia-containing structures. We hypothesize that HIP and high-fat diet influence milk oligosaccharide composition, particularly sialylated oligosaccharides. Furthermore, we propose that milk Sia content influences pup brain Sia content. To test these hypotheses we (i) characterize mouse milk oligosaccharides and Sia concentrations in mouse milk of a GDM mouse model with dietary fat intake intervention; and (ii) determine Sia levels in offspring brains.

The concentrations of oligosaccharides and Sia in mouse milk and offspring's brains were quantified using UPLC-FLD analysis. Analyses were performed on surplus samples from a previous study, where HIP was induced by combining high-fat diet (HF) feeding and low-dose streptozotocin injections in C57Bl/6NTac female mice. The previous study described the metabolic effects of HIP on dams and offspring.

We detected 21 mouse milk oligosaccharides, including 9 neutral and 12 acidic structures using UPLC-MS. A total of 8 structures could be quantified using UPLC-FLD. Maternal HIP and HF diet during lactation influenced sialylated oligosaccharide concentrations in mouse milk and total and free sialic acid concentrations. Sia content in offspring brain was associated with total and free Neu5Gc in mouse milk of dams, but no correlations with HIP or maternal diet were observed.

## Graphical abstract

**Figure:**
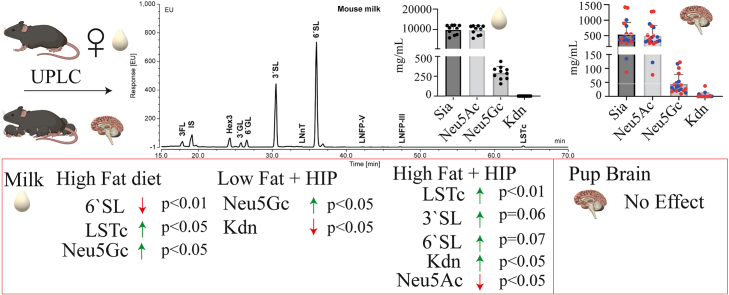

## 1 Introduction

Breast milk is considered an optimal feeding source for newborns and contains various nutrients and biological active factors important for growth and development [[1], [2]]. Human milk oligosaccharides (hMOS) are human milk's most abundant bioactive factors, with concentrations of more than 20 g/L in colostrum and around 7–15 g/L in mature milk [3]. The structures of hMOS are complex and more than 160 hMOS have been structurally characterized [[4], [5], [6]]. Human milk oligosaccharides play a fundamental role in intestinal microbiome establishment and balance, infection prevention, immune system maturity, and brain development in the neonate [7]. The content of oligosaccharides in milk from other mammals shows far less quantity and variety of oligosaccharides than in human milk [[8], [9], [10]]. Moreover, sialylated oligosaccharides are more abundant in non-human milk [9,11].

Sialic acids (Sia) are important residues of sialylated oligosaccharides in breast milk [12]. Sia are also found in the poly Sia chain and gangliosides, which are crucial for brain function. Sia are also found as free monosaccharides in body fluids like plasma, urine, saliva, and breast milk. There are three main representative Sia forms, including *N*-acetylneuraminic acids (Neu5Ac), *N*-glycolylneuraminic acids (Neu5Gc), and 2-keto-3-deoxynonulosonic acid (Kdn) [12]. Due to a population-wide mutation in the gene for CMP-Neu5Ac hydroxylase (CMAH), humans cannot synthesize CMP-Neu5Gc. In all other animals, Neu5Gc occurs in varying levels. Human glycosylation can contain minor amounts of Neu5Gc through incorporation from the diet. Kdn is the newest discovered member of the Sia family, which is present at lower levels than Neu5Ac and Neu5Gc in most mammalian tissues [13]. Notably, recent evidence suggests that dietary Sia might alter Sia concentrations in the brain [14] and thus may play a key role in the development of cognition and learning ability [15]. Some studies have associated milk sialic acid, particularly from sialylated milk oligosaccharides, with cognitive development in mouse and rat studies [12,[16], [17], [18], [19]].

The mouse is widely used as an experimental model in scientific research, however, very little is known about mouse milk composition. To date, to the best of our knowledge, there are only three studies in which mouse milk oligosaccharides (mMOS) have been reported with limitations [11,20,21]. Prieto et al. (1995) have identified 3-fucosyllactose (3FL), 3′-sialyllactose (3′SL), and 6′-sialyllactose (6′SL) in mouse milk [21]. Fuhrer et al. (2010) focused on concentrations of 3′SL and 6′SL across the course of lactation in mice [20]. Li et al. (2021) studied only acidic oligosaccharides in rat and mouse milk, with 12 structures reported for the first time [11]. However, the absolute concentrations of oligosaccharides and profiles of neutral and acidic oligosaccharides in mouse milk have not been studied.

Only one study reported total Sia concentrations in mouse milk [22] and one study reported that Neu5Gc accounts for 30 % of the Sia amount in mouse milk [23]. However, the total and free Neu5Ac and Neu5Gc concentrations in mouse milk are still unknown. Additionally, several studies report total Sia levels in mouse pup brains, but reported concentrations of total Sia range from 445 to 37,830 ng/mg [[24], [25], [26]]. It is unknown whether these concentrations are related to the health or metabolic status of the mother.

Hyperglycemia in pregnancy (HIP) has considerable adverse effects on mother and infant, increasing the risk of obesity, type 2 diabetes, and cardiovascular disease later in life [27,28]. There are different types of HIP, including pre-gestational diabetes mellitus (PDM), gestational diabetes mellitus (GDM), and diabetes in pregnancy (DIP) [29]. PDM occurs in women who have been diagnosed with Type 1 or Type 2 diabetes before pregnancy. GDM first appears during pregnancy and leads to milder and usually transient gestational hyperglycemia. DIP concerns women who have been diagnosed with Type 1 or Type 2 diabetes in early pregnancy. It has been reported that a higher carbohydrate content is present in the colostrum of GDM mothers [30]. The total Sia concentration in serum appears to be significantly higher in women with GDM than those without GDM [31]. Recently, one study reported significantly higher amounts of fucosylated and sialylated *N*-glycans in a lean GDM model mouse milk [32]. There is however no detailed study of the effects of HIP on milk oligosaccharides and Sia concentration in mouse milk.

We hypothesize that HIP can induce systemic, low-grade inflammation, which influences the levels of Sia in circulation and milk. Similarly, HF diets have been shown to influence Sia levels in serum and milk. We further propose that altered Sia levels in milk may influence brain Sia levels. In the current study, we made use of a lean GDM mouse model that combines multiple low-dose streptozotocin (STZ) injections prior to pregnancy with short-term, high-fat (HF)-feeding as previously described [3]. Other lean mouse models use STZ injections during pregnancy [32]. This approach results in insulin-sensitive, lean GDM dams [3]. In this study, we aimed to (i) characterize quantitative profiles of mMOS and Sia concentrations in mouse milk and the offspring brain; (ii) identify whether maternal HIP (PDM/GDM) affects the bioactive components in mouse milk (oligosaccharides and Sia) and whether that influences Sia content in the brain of offspring.

## 2 Material and methods

### 2.1 Materials

Quantitative oligosaccharide standards 2′-fucosyllactose (2′FL), 3-fucosyllactose (3FL), 3′-silayllactose (3′SL), lacto-*N*-tetraose (LNT), lacto-*N*-*neo*tetraose (LNnT), lacto-*N*-fucopentaose I (LNFP I), and lacto-*N*-difocohexaose I (LND I) and qualitative standard sialyllacto-*N*-tetraose a (LSTa), were obtained from Elicityl Oligotech (Crolles, France). Quantitative standards β-3′-galactosyllactose (3′GL), β-4′-galactosyllactose (4′GL), β-6′-galactosyllactose (6′GL), 6′-sialyllactose (6′SL), 5-*N*-acetylneuraminic acid (Neu5Ac), 5-*N*-glycolylneuramicic acid (Neu5Gc) and keto-deoxy-neuraminic acid (Kdn) and qualitative standard lacto-*N*-fucopentaose V (LNFP V) were obtained from Carbosynth/Biosynth B.V. (Lelystad, the Netherlands). Quantitative standards lacto-*N*-fucopetaose II (LNFP II), lacto-*N*-fucopentaose III (LNFP III), disialyllacto-*N*-tetraose (DLSNT), and difucosyllactose (DFL), as well as qualitative standards difucosyllacto-*N*-hexaose a (DFLNHa), siayllacto-*N*-tetraose b (LSTb), sialyllacto-*N*-tetraose c (LSTc), 3′-sialyl-3-fucosyllactose (3′ S3FL) and difucosyllacto-*N*-*neo*hexaose (DFLNnH) were obtained from IsoSep ab (Tullinge, Sweden). Qualitative standards for lacto-*N*-fucopentaose VI (LNFP VI) and monofucosyllacto-*N*-hexaose III (MFLNH III) were obtained from Dextra Laboratories UK (Reading, United Kingdom). Laminaritriose internal standard was obtained from Megazyme (Wicklow, Ireland) and maltotriose calibration standard was obtained from Merck Netherlands BV (Amsterdam, the Netherlands). Nestlé Research kindly provided a human milk quality control (QC) sample to validate milk oligosaccharide results.

### 2.2 Experimental design and sample collection

**Fig. 1:**
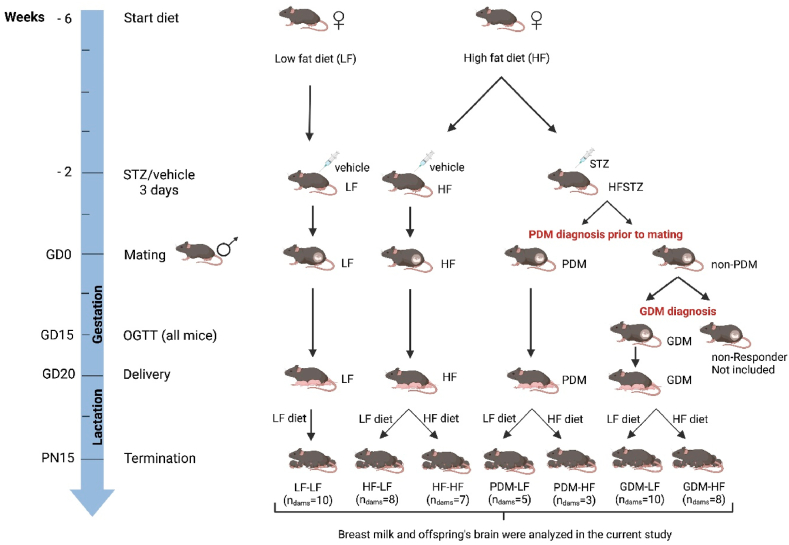
Animals study design. Seven groups were developed in this study, including LF, HF-LF, HF–HF, PDM-LF, PDM-HF, GDM-LF, and GDM-HF. In total, mouse milk (n = 51) was collected from dams (n = 51) and the brain tissue (n = 121) was collected from offspring (n = 121). There are 7 GDM-LF and 7 GDM-HF dams recovered at PN 15.

All mouse samples (milk and brain tissue) were left-over samples from a previous study, which was conducted following institutional guidelines for the care and use of laboratory animals at the University of Groningen [3]. All animal procedures related to the purpose of the research were approved by the local Animal Welfare Body under an Ethical license provided by the national competent authority (Centrale Commissie Dierproeven, CCD, #AVD1050020185445), securing full compliance with the European Directive 2010/63/EU for the use of animals for scientific purposes. The details of the experimental setup, including the breeding protocol, power calculations, and drop-outs of the mouse study are described in the original study [3]. Summarized, as shown in Fig. 1, 9-week-old female C57Bl/6NTac mice (Taconic, Denmark) were housed in pairs and 8-week-old male C57Bl/6NTac mice (Taconic, Denmark) were housed individually to avoid fighting. Females were fed a 10 % low-fat diet (LF; D12450Ji, Research Diets) or a 60 % high-fat diet (HF, D12492i, Research Diets), while male breeders were fed a chow diet (RMH-B, AB-diets, Woerden, The Netherlands). All mice were kept under a 12-h light-dark cycle (7:00–19:00). After 4 weeks of diet, female mice received intraperitoneal injections with either 60 mg/kg STZ (S0130, Sigma-Aldrich) or vehicle for three consecutive days and were screened for PDM prior to mating and classified as such if random blood glucose (RBG) > 12 mmol/L at gestational day (GD) 0. After mating, all pregnant dams underwent an oral glucose tolerance test (OGTT) on GD 15 as described [3]. Dams that were normoglycemic at GD 0 were diagnosed with GDM if the 2 h OGTT BG was >12 mmol/L at GD 15. RBG levels on postnatal day (PN) 15 were used to assess GDM recovery, defined as RBG <12 mmol/L. To discriminate the effects of direct HF exposure during lactation, half the dams in the HF, GDM, and PDM groups were transferred to an LF diet on PN2 (Fig. 1).

Prior to termination at PN15, dams were transferred to a new cage to separate them from their pups for 2 h to allow for sufficient milk in the mammary gland. Then dams were anesthetized with ketamine/dexmedetomidine i.p. and received Oxytocin i.p. (1IU, Synthocinon®, Sigma-Tau Industrie Farmaceutische Riunite). Dams were milked in a random order using a modified human electric breast pump (Calypso, Ardo Medical AG, Unterägeri, Switzerland) for up to 15 min or until 500 μL of milk was collected [33]. Milk samples were kept at −80 °C for further analysis. In the original study, the anesthetized dams were terminated right after milking by cardiac puncture [34]. Blood was centrifuged (4000 rpm for 10 min at 4 °C) and plasma was stored at −80 °C for analysis. Pups were anesthetized with isoflurane and terminated by cardiac puncture. Tissues from dams and pups were rapidly excised and either snap-frozen or fixed in 4 % (wt/vol) formaldehyde in PBS. Samples were stored at −80 °C for less than 12 months before the current study. It should be noted that no new animals were used in the current study, only stored samples from the previous study were re-used [3].

### 2.3 Human and mouse milk oligosaccharides analysis

Human/mouse milk oligosaccharides were labeled with 2-aminobenzamide (2-AB) and separated by UHPLC-FLD according to previously described methods [35]. In short, 20 μL human/mouse milk was mixed with the same volume of internal standard (laminaritriose, 0.5 μmol/mL). A total 20 μL of each mixture was transferred to a new microtube and the labeling solution was added (2-AB [0.35 M] + 2-picoline borane complex [1.0 M] in dimethylsulfoxide containing acetic acid [30 %], 200 μL). The solution was placed in a water bath at 65 °C for 2 h. After cooling for 15 min at 4 °C, 600 μL 75 % acetonitrile was added. The labeled oligosaccharides were separated and quantified by UHPLC-FLD using an H-class Waters UHPLC system (Waters Chromatography B.V., Etten-Leur, The Netherlands), equipped with a guard column (BEH 5 mm) between switching valves, to allow on-line sample cleanup. All oligosaccharides were quantified against a maltotriose calibration curve with known purity, assuming equimolar response factors. The limits of detection (LoD) and quantification (LoQ) of milk oligosaccharides were determined in accordance with previous studies [35]. The analytical system was validated using real HMO standard curves and a human milk QC sample to ensure batch-to-batch reproducibility.

### 2.4 Milk oligosaccharides purification and mass spectrometry analysis

The labeled oligosaccharides were purified using a porous graphitized carbon (PGC) solid phase extraction (SPE) column pre-activated and conditioned with acetonitrile and water. The SPE column was initially activated by 80 % acetonitrile mixed with 0.1 % trifluoroacetic acid (TFA) (1 mL, three times). Milli-Q water mixed with 0.05 % TFA was used to condition the column (1 mL, thrice). Then, samples of labeled oligosaccharides (0.5 mL) were loaded on the column. After washing with Milli-Q water mixed with 0.1 % TFA (1 mL, three times), the oligosaccharides were eluted with 40 % acetonitrile mixed with 0.1 % TFA (1 mL, three times). The 3 mL elution fractions from the PGC SPE were collected and dried to 1 mL under nitrogen (N_2_) for LC-MS analysis of individual mMOS.

The LC-MS analyses were established using an HPLC instrument (Shimadzu 20 series) followed by an Orbitrap Velos Pro high-resolution mass spectrometer (Thermo-Fisher Scientific, USA). The HPLC system consisted of an Acquity BEH Glycan (1.7 mm, 2.1 × 150 mm), VanGuard BEH amide (1.7 mm, 2.1 × 50 mm) (Waters, The Netherlands) and a 6-port valve. Samples were injected (25 μL) and separated at 55 °C with a gradient (0.35 mL/min) of 50 mM ammonium formate (solvent A) and acetonitrile (solvent B). The gradient program started with 5 % A for 2.5 min, then ramped %A from 5 % to 10 % for 2.5 min, then 10 %–19 % in 27.5 min, 19–22 % in 16.0 min, 22 %–27 % in 13.4 min and 22 %–32 % in 27.5 min. After eluting the oligosaccharides, the column was washed with 60 % A for 2.0 min and re-equilibrated with 10 % A for 5.0 min. MS acquisition was performed in a positive electrospray mode from *m/z* 200 to 2000 at a spray voltage of 3.5 kV and a resolution of 60,000. The source temperature was 325 °C and the ion transfer tube temperature was 350 °C. Data were analyzed by MZmine 3 software.

### 2.5 Sialic acids analysis in mouse milk

Sialic acids in mouse milk were derivatized by 1,2-diamino-4,5-methylenedioxybenzene (DMB) and then were analyzed by UHPLC-FLD. The milk sample was diluted 100 times using Milli-Q water. 20 μL of diluted samples were mixed with the same volume of 1 M formic acid (total Sia) and hydrolyzed for 1 h at 85 °C. For total Sia, the hydrolyzed solution was mixed with 40 μL DMB reagent (7 mM DMB, 1.4 M acetic acid, 18 mM sodium hydrosulfite, 0.75 M 2-mercaptoethanol) and heated at 85 °C for 50 min. For free Sia, diluted samples were mixed with 20 μL Milli-Q water and 40 μL DMB reagent (7 mM DMB, 40 mM TFA, 18 mM sodium hydrosulfite, 1 M 2-mercaptoethanol) and kept at 4 °C for 48 h. The derivatized samples were then cooled to room temperature and diluted with 150 μL Milli-Q water. Afterwards, Sia were separated by UHPLC-FLD. Quantitation was achieved against calibration curves of Neu5Ac, Neu5Gc, and Kdn (0.005–2.000 nmol/μL).

### 2.6 Total sialic acids analysis in mouse pup brain

Brain tissue was homogenized with PBS (20 μL/mg) using pellet pestles. The whole mixture was centrifuged at 13,000×*g* for 30 min. The homogenized sample (20 μL) was mixed with the same volume of 1 M formic acid and hydrolyzed for 3 h at 85 °C. The hydrolyzed solution was mixed with 40 μL DMB reagent (7 mM DMB, 1.4 M acetic acid, 18 mM sodium hydrosulfite, 0.75 M 2-mercaptoethanol) and heated at 85 °C for 50 min. The derivatized sample was then cooled to room temperature and diluted by adding Milli-Q water (150 μL). Then Sia were separated by UHPLC-FLD.

### 2.7 Liver and plasma analysis

Lipid contents of dam liver samples, including triglycerides and cholesterol, were determined by commercially available kits (Roche Diagnostics) after lipid extraction. Alanine aminotransferase (ALT) activity of dam blood plasma was analyzed by a Cobas 6000 analyzer with standard reagents (Roche Diagnostics).

### 2.8 Data analysis

Sample size calculations and drop-outs are described in detail in the original study, the current study re-uses leftover materials and existing data [3]. All figures were created using GraphPad Prism (Version 9) and all statistical analyses were performed by SPSS (Version 23). The concentrations of oligosaccharide and Sia are presented as mean ± Standard Deviation (SD). When data were normally distributed, an unpaired *t*-test or one-way ANOVA with Tukey's multiple comparison test was used for group comparisons; when data were non-normally distributed, a nonparametric test or Kruskal-Wallis with Dunn's multiple comparison test was used. Correlations within the milk Sia and brain Sia, the milk content (oligosaccharides and Sia), and physiological parameters (maternal and pup) were assessed using Spearman's correlation coefficient. *P* values < 0.05 were considered statistically significant. In case significant correlations were observed between milk composition and maternal factors, we checked if those maternal factors significantly differed between groups and corrected for this where necessary [3].

## 3 Results and discussion

### 3.1 Oligosaccharide profiles in mouse milk

**Fig. 2:**
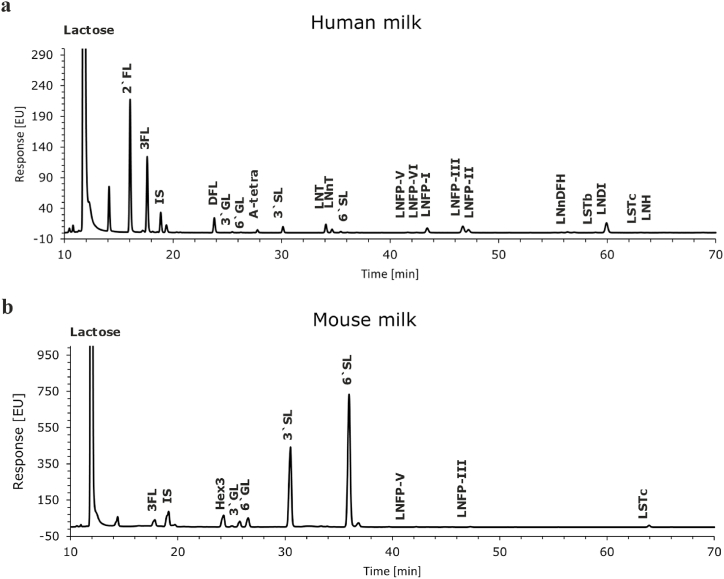
UHPLC profiles of oligosaccharides in (a) human milk. (b) Mouse milk.

To characterize the profile of the mouse milk oligosaccharides, we analyzed the oligosaccharides in dam mature milk collected in a previous study at postnatal day 15 (PN15) [3]. The oligosaccharide profiles of human (QC sample) and mouse milk are shown in Fig. 2a and b, respectively. By using a QC human milk sample that is also used in human milk studies [35] and infant nutrition analysis [36], we ensure batch-to-batch reproducibility. In total, 4 fucosylated (3FL, LNFP-V, LNFP-III, LNnDFH), 5 neutral (Hex3, 3′GL, 6′GL, NAL, LNnT), and 12 sialylated oligosaccharides (6Su-L, 6Su-GL, 3′SL, 6′SL, 3′NGL, 6′NGL, SLN, S-GL, S-GLN, NG-SL, LSTc, NG-NADHL) were identified in mouse milk by LC-MS (S-Table 1). LC-MS data confirmed Hex3 confirmed the tri-hexose structure of Hex3. The elution position did not correspond with 3′-GL, 4′-GL, or 6′-GL. Structure Hex3 is possibly α3′-galactosyllactose, identified previously in some non-human milk [10,37]. Among oligosaccharides in mouse milk, we identified 10 oligosaccharide structures, including 3FL, LNFP-V, LNFP-III, LNnDFH, 3′GL, 6′GL, LNnT, 3′SL, 6′SL, and LSTc, that were also present in human milk (Fig. 2). Hex3, NAL, 6Su-L, 6Su-GL, 3′NGL, 6′NGL, SLN, S-GL, S-GLN, NG-SL, and NG-NADHL were exclusively found in mouse milk (Fig. 2). To our best knowledge, 8 neutral oligosaccharides (LNFP-V, LNFP-III, LNnDFH, Hex3, 3′GL, 6′GL, NAL, LNnT) were not described in mouse milk before.

As shown in S-Table 2 and S-Table 3, the concentrations of 3FL, Hex3, 3′GL, 6′GL, LNFP-III, 6′SL, 3′SL, and LSTc were above limits of quantification (LoQ) in mouse milk. 6′SL and 3′SL were the dominant oligosaccharides in mouse milk, which aligns with previous studies [20,21]. The LC-MS analysis also showed evidence of trace amounts of two isomers of Neu5Gc decorated lactose, but no peaks above LoD were observed in the UPLC-FLD chromatogram. 6′SL showed the highest concentration in mouse milk, with a mean concentration of 3728 mg/L in the LF control group. 3′SL was the second dominant oligosaccharide in mouse milk (1332 mg/L). Fuhrer et al. (2010) detected the concentrations of 6′SL and 3′SL across lactation from milk in wild-type mice. This study showed that 3′SL was dominant in the first week of lactation, while 6′SL levels showed a small rise in the middle of lactation and became the most abundant at PN15 [20]. However, they reported the concentrations in mg/100 g of milk solids. Assuming ∼40 % milk solids [38], the concentrations of 6′SL and 3′SL (∼2800 and 2000 mg/L) around PN15 in that study [20], are in a similar range as our data. A small amount of LSTc (31 mg/L) was detected in mouse milk in our study, which is in accordance with the study of Li et al. (2021), who identified 6′SL, 3′SL, and LSTc as the three dominant acidic oligosaccharides in mouse milk [11]. Moreover, 3FL (∼79 mg/L) was also found in mouse milk, consistent with a previous study that showed a trace of 3FL (∼5 mg/L) in wild-type mouse milk, using a different analytical approach (postnatal days 5–12) [21]. In addition, the same content of 3′GL and 6′GL (∼25 mg/L), Hex3 (15 mg/L), as well as LNFP-III (16 mg/L) were found in mouse milk. It should be noted that in most mice the concentration of Hex3 is below LoQ (n = 36). In contrast, most of the remaining mice (n = 13) have concentrations of Hex3 between 7 and 25 mg/L, and finally two mice have significantly higher Hex3 concentrations, i.e. 47 and 266 mg/L, respectively. To the best of our knowledge, this is the first time that the concentrations of 3′GL, 6′GL, Hex3, and LNFP-III in mouse milk are being reported.

### 3.2 Maternal HIP or HF exposure during lactation affects sialylated oligosaccharide concentrations in mouse milk

To identify whether maternal HIP (PDM and GDM) or maternal diet during lactation influences the oligosaccharide concentrations, samples from six groups from a previous study were re-used in our study, i.e., HF-LF, HF–HF, GDM-LF, GDM-HF, PDM-LF and PDM-HF (Fig. 1) [3].

**Fig. 3:**
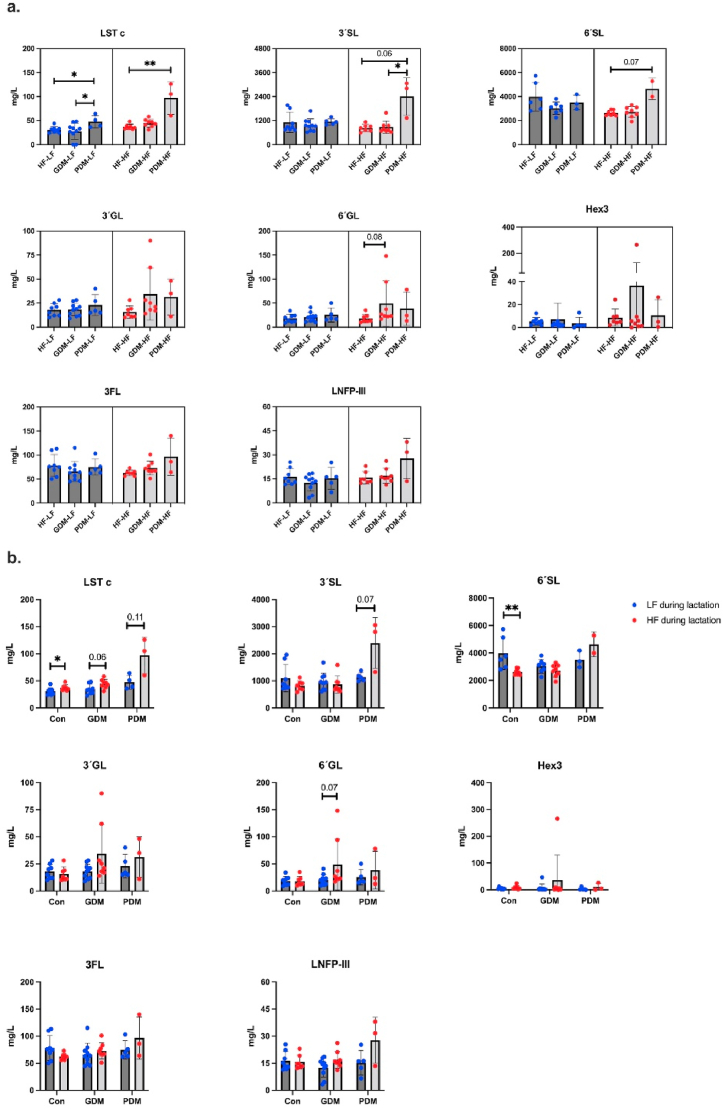
(a) The concentration of oligosaccharides in LF (HF-LF, GDM-LF, PDM-LF) and HF (HF–HF, GDM-HF, PDM-HF) groups. (b) The concentration of oligosaccharides in control (LF, HF), GDM (LF, HF), and PDM (LF, HF) groups. Blue points = low-fat diet during lactation, red points = high-fat diet during lactation. One-way ANOVA followed by Tukey's multiple comparison test (LF) or Kruskal Wallis followed by Dunn's multiple comparison test (HF) were used. *p < 0.05, **p < 0.01.

Firstly, to understand whether maternal HIP (PDM and/or GDM) affects milk oligosaccharide concentrations, we classified all subgroups that switched to a low-fat diet at the start of lactation as LF [HF-LF, GDM-LF, PDM-LF] group and all groups that continued on the high-fat diet as HF [HF–HF, GDM-HF, PDM-HF] group (Fig. 3a). When mice were switched to a low-fat diet (LF) during lactation, mouse milk in PDM-LF contained an higher level of LSTc than that in GDM-LF (*p* < 0.05) and HF-LF (*p* < 0.05). This may suggest that LSTc is influenced by deviations in glucose metabolism, which persisted in the PDM animals but were transient in most GDM animals.

When mice continued to receive a high-fat diet (HF) during lactation, milk from PDM-HF mice showed a higher amount of LSTc (97 mg/L), 3′SL (2397 mg/L), and 6′SL (4631 mg/L), compared to HF–HF control mice (*p* < 0.01, *p* = 0.06, *p* = 0.07, respectively). Although the sample size of the PDM-HF group was limited (n = 3), the concentration of LSTc, 3′SL, and 6′SL for each mouse was consistently higher than in HF–HF mice. Moreover, the mouse milk in PDM-HF mice also contained a significantly higher level of 3′SL compared to GDM-HF mice (*p* < 0.05). Previous human studies have reported that the 3′SL is elevated in the maternal serum of GDM women at pregnancy week 15 [39] Women with GDM also had elevated levels of 3′SL in cord blood compared with healthy mothers [40]. It is known that inflammation results in higher levels of protein *N*-glycan sialylation [41,42]. Possibly systemic inflammation results in higher 3′SL levels. Although the 3′SL concentration was not higher in milk from GDM dams. PDM combined with a high-fat diet during lactation may induce more inflammation, resulting in the highest level of 3′SL in PDM-HF mouse milk. Our results showed that PDM significantly affects the sialylated oligosaccharide concentrations but not neutral oligosaccharides in mouse milk. Moreover, no statistically significant differences were observed in the concentration of sialylated milk oligosaccharides between HF-LF, HF–HF, GDM-LF, and GDM-HF mice in our study, suggesting that high-fat diet alone does not significantly influence milk oligosaccharide concentrations. Still, in case of PDM, there is a difference between LF and HF diet during lactation. Our results fit with previous findings [32], which showed no differences in milk sialylated oligosaccharides (3′SL, 6′SL, LSTc) were found between mouse dams with or without GDM. Notably, 70 % of the GDM-LF and 88 % of the GDM-HF dams had recovered to normal glucose metabolism by the time the milk was collected. Thus, the milk collected at PN15 may relate to a time point at which inflammation had resolved in GDM dams, explaining why 3′SL concentrations were not changed compared to controls. Future studies should include milk sampling at earlier time points to assess whether GDM influences early-stage milk composition.

The Spearman rank correlation coefficient (r) between mMOS concentrations and maternal parameters, which were described previously [3], are shown in Supplemental Table 4a-e (control, GDM, PDM, HIP). Maternal parameters include body weight, gestational weight, lactation weight gain, glucose level, liver weight and lipid (triglyceride and cholesterol) content, and plasma alanine aminotransferase (ALT) data from the original study [3]. As illustrated in Supplemental Tables 4 and 3′SL in the PDM group (PDM-LF and PDM-HF) showed strong positive correlations with maternal body weight at PN15 (r = 0.87, *p* < 0.05). The 3FL, 3′GL, and 6′GL concentrations were mildly positively correlated with lactation weight gain in PDM groups. On the contrary, in the HF-LF and HF–HF groups, 3FL, 3′GL, and 6′GL presented negative correlations with lactation weight gain (difference between body weight at PN15 and PN2). When evaluated across the study population no correlations were observed with lactation weight gain. Moreover, in the GDM group, 3FL, 6′GL, LST c, and LNFP-III were significantly associated with liver triglyceride levels (r = 0.56, 0.48, 0.51, 0.51). In the PDM group, 3′SL was positively associated with plasma ALT (r = 0.90, *p* < 0.05), while LSTc and LNFP-III were negatively associated with plasma ALT (r = −0.90 and −0.75, *p* < 0.05). However, the correlations between 3′SL and plasma ALT were weak in the HIP group. It should be noted that LNFP-III was mostly detected at concentrations close to the LoQ for the analytical method and the significance of correlations should therefore be considered carefully. Plasma ALT activity has been regarded as a marker of liver disease and might hence more generally reflects metabolic syndrome [34]. Both plasma ALT and systemic inflammation are related to metabolic disease and plasma ALT is correlated with 3′SL in our study. This finding may further support the hypothesis that 3′SL in mouse milk is influenced by maternal systemic inflammation.

Next, to identify whether exposure to a high-fat diet during lactation affects milk oligosaccharide concentrations, HF-LF vs. HF–HF, GDM-LF vs. GDM-HF, and PDM-LF vs. PDM-HF groups were compared respectively (Fig. 3b). Compared to HF-LF mice, the LSTc level in milk was significantly increased in HF–HF mice (*p* < 0.05). The same trends were also observed in the GDM (*p* = 0.06) and PDM (*p* = 0.11), yet failed to reach statistical significance. Similarly, milk 3′SL showed a trend towards higher concentration in PDM-HF compared to PDM-LF (*p* = 0.07). On the contrary, the concentration of 6′SL in HF–HF milk was significantly decreased (*p* < 0.01) compared to HF-LF milk. Our results suggest that exposure to a high-fat diet during lactation significantly affects sialylated milk oligosaccharides. Since 6′SL is the dominant oligosaccharide in mouse milk, our data align with human studies, which showed a high-fat diet during lactation to be linked to a decrease in sialylated hMOS [43]. In addition, the total oligosaccharide concentrations were comparable between LF-LF and HF-LF groups, suggesting that exposure to a high-fat diet during gestation does not influence the oligosaccharide content in mouse milk collected at PN15 (Supplemental Figure 1).

The Spearman rank correlation coefficients (r) between mMOS concentrations and maternal parameters are shown in Supplemental Tables 4d and 4e (LF, HF). There are very few correlations between mMOS and maternal factors. The mice receiving LF diet during lactation (S-Table 4d) showed only medium strength significant correlations between 3′SL (r = 0.47, p < 0.05) and LSTc (r = 0.48, p < 0.05) with PN15 glucose and between LNFP-III and gestational weight gain (r = −0.42, p < 0.05) and lactational weight gain (r = 0.48, p < 0.05). It should be noted that LNFP-III is detected close to LoQ levels. In HF groups (HF–HF, GDM-HF and PDM-HF; S-Table 4e), Only medium strength correlations were observed with significance for 3FL with liver triglycerides (r = 0.56, p < 0.05), for 3′SL with PN15 glucose (r = 0.56, p < 0.05) and LSTc with liver triglycerides (r = 0.60, p < 0.05) and liver total cholesterol (r = 0.59, p < 0.05).

### 3.3 Sialic acid concentrations in mouse milk

**Fig. 4:**
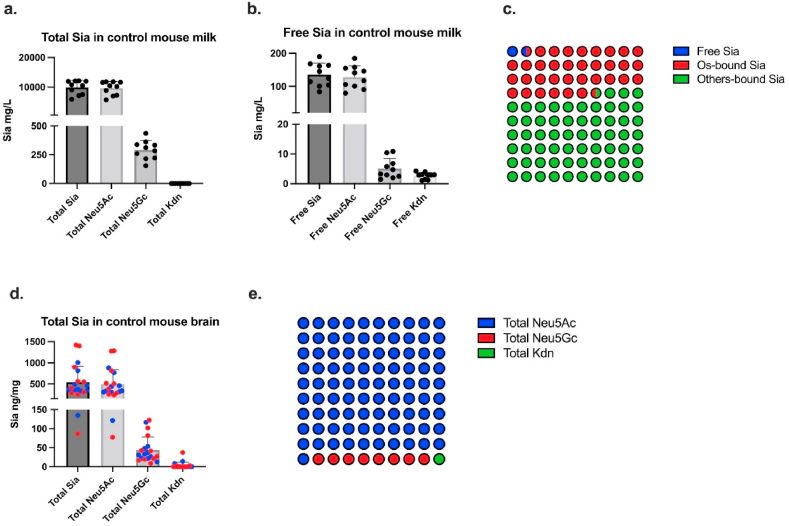
(a) The concentration of total Sia (Neu5Ac, Neu5Gc, Kdn) in mouse milk. (b) The concentration of free Sia (Neu5Ac, Neu5Gc, Kdn) in mouse milk. (c) The percentage of free and bound Sia in total milk Sia. Os, oligosaccharides. (d) The concentration of total Sia (Neu5Ac, Neu5Gc, Kdn) in mouse brain. Blue points = male pups, red points = female pups. (e) The percentage of total Neu5Ac, Neu5Gc, and Kdn in mouse brain Sia.

We quantified Sia levels in mouse milk at PN15. The concentrations of total and free Sia (Neu5Ac, Neu5Gc, and Kdn) in mouse mature milk are shown in Fig. 4, S-Table 3, and S-Table 5. The mean concentration of total Sia was 9918 mg/L in the control group (LF-LF), including Neu5Ac (9626 mg/L), Neu5Gc (292 mg/L) and limited Kdn (0.08 mg/L) (Fig. 4a). These concentrations are slightly higher than Dickson and Messer (1978) reported earlier, who detected a total Sia concentration of 9278 and 4948 mg/L in mouse milk (BALB-C) at PN8 and PN11, respectively. Their study used 0.1 M H_2_SO_4_ at 85 °C for 20 min to release Sia and determined its levels using thiobarbituric acid. However, only two mouse milk samples were analyzed in that study while the mouse strain (BALB-C) differed from ours (C57Bl/6NTac). Moreover, our data showed that more than 96 % of total Sia in mouse milk were Neu5Ac, while Neu5Gc only comprised 1–4 % of total Sia. There is one previous study that reports 30 % Neu5Gc in mouse milk (C57Bl/6), however, due to a lack of internal standard, Neu5Gc could only be quantified relative to the Neu5Ac amount in that study [23]. In addition, the free Sia level was 136 mg/L in control mice (LF-LF), including free Neu5Ac 128 mg/L, free Neu5Gc 5 mg/L and free Kdn 3 mg/L (Fig. 4b). This is the first report of free Sia in mouse milk. Among Sia in mouse milk, free Sia only accounted for 1.4 % of total Sia, oligosaccharide-bound Sia accounted for 35.2 %, and the rest of Sia is likely bound to proteins or lipids (Fig. 4c). Previous reports have shown a high rate of sialylation in mouse milk protein *N*-linked glycans, which was elevated by GDM [32]. Mouse milk glycolipid content has not been studied in detail, but human and bovine milk contains ∼9 g/L and ∼4 g/L gangliosides (sialylated glycolipids), respectively [44]. Depending on the balance of predominant mono- or disialylated forms, between 18 and 31 % of the ganglioside weight consists of sialic acid, potentially contributing significantly to the milk's bound sialic acid fraction.

### 3.4 Maternal HIP and continued HF diet during lactation influence the Neu5Gc concentration in mouse milk

**Fig. 5:**
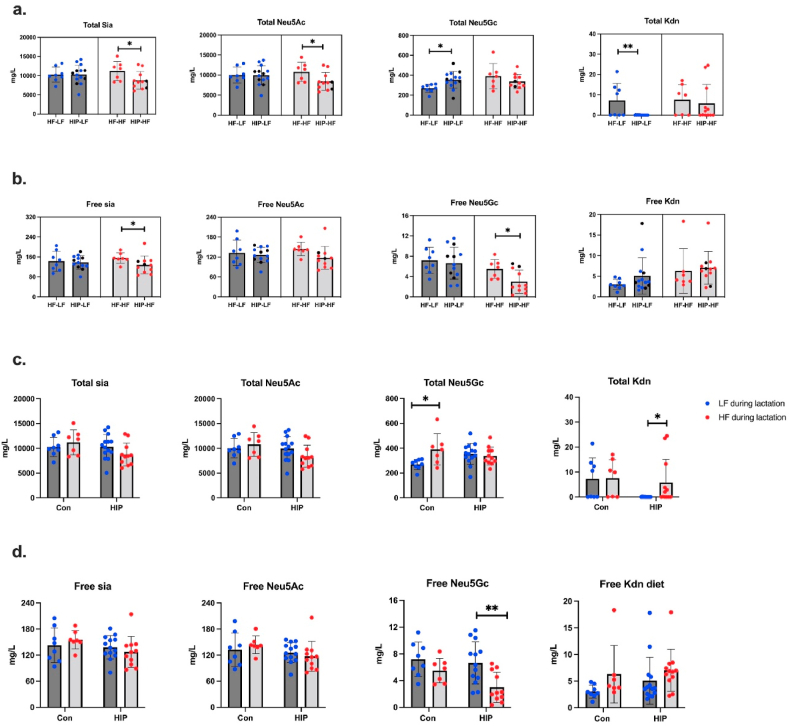
(a) The concentration of total milk Sia (Neu5Ac, Neu5Gc, Kdn) in LF (HF-LF, HIP-LF) and HF (HF–HF, HIP–HF) groups. (b) The concentration of free milk Sia (Neu5Ac, Neu5Gc, Kdn) in LF (HF-LF, HIP-LF) and HF (HF–HF, HIP–HF) groups. (c) The concentration of total milk Sia in control (LF, HF) and HIP (LF, HF) groups. (d) The concentration of free milk Sia in control (LF, HF) and HIP (LF, HF) groups. Blue points = low-fat diet during lactation, red points = high-fat diet during lactation. Black points = PDM mice. One-way ANOVA followed by Tukey's multiple comparison test (Total Sia, Neu5Ac, Neu5Gc) or Kruskal Wallis followed by Dunn's multiple comparison test (Free Sia, Neu5Ac, Neu5Gc, Kdn, total Kdn) were used. *p < 0.05, **p < 0.01.

To characterize whether maternal HIP (GDM and/or PDM) or high-fat diet during lactation influenced Sia content in mouse milk, we compared four groups, i.e., HF-LF, HF–HF, HIP-LF, and HIP–HF (Fig. 5). Considering the low sample size of the PDM group, and a lack of significant differences in Sia concentrations between GDM and PDM mice, we combined GDM and PDM into a single HIP group (Supplemental Figure 2).

When HIP dams were switched to an LF diet during lactation, they showed a significantly higher level of total Neu5Gc in milk (*p* < 0.05) compared to HF-LF mice (Fig. 5a and b). The HIP-LF mice showed a significantly lower total Kdn (p < 0.01) level than HF-LF mice. However, there were no significant differences in total milk Sia and Neu5Ac between HF-LF and HIP dams on the LF diet. On the contrary, when mice continued to receive HF diet during lactation, the total Sia, Neu5Ac, free Sia, and free Neu5Gc levels were decreased in HIP–HF mice (*p* < 0.05) (Fig. 5a and b). These findings suggest that a combination of maternal HIP and continued HF diet during lactation influences milk Sia contents.

Subsequently, we evaluated whether direct exposure to HF diet during lactation influences Sia content in mouse milk (Fig. 5c and d). Compared to HF-LF mice, HF diet during lactation significantly increased the total Neu5Gc level in milk from HF–HF mice (*p* < 0.05) (Fig. 5c). In the HIP group, the HF diet during lactation significantly increased the total Kdn concentration (*p* < 0.05). However, Kdn was present in levels close to the LoQ and correlations should be interpreted carefully. The free Neu5Gc level was significantly decreased by HF feeding (*p* < 0.01). These results again suggest that HF diet during pregnancy and lactation affects milk Neu5Gc concentration. However, as no significant differences were found in milk Sia between LF-LF and HF-LF groups (Supplemental Figure 3), HF feeding selectively during gestation does not appear to influence milk total Sia levels.

Spearman rank correlation coefficients (r) between milk Sia concentrations and maternal parameters are shown in Supplemental Table 7 (HF-LF, HF–HF, HIP-LF, HIP–HF). In HF-LF and HF–HF groups, total Nue5Gc significantly correlated with lactation weight gain (r = 0.57, *p* < 0.05). Previously, it was found that in this mouse population, the lactation weight gain was not different between control, GDM, or PDM mice [3]. On the contrary, in the HIP-LF and HIP–HF groups (Supplemental Table 7b), total Sia (r = −0.50, *p* < 0.05), Neu5Ac (r = −0.50, *p* < 0.05) and Neu5Gc (r = −0.40, *p* < 0.05) showed negative correlations with lactation weight gain. Free milk Kdn concentrations were very low, close to LoQ, however, Spearman rank correlations showed significant correlations with liver markers in mice that continued on an HF diet during lactation (Supplemental Table 7d). Whether this is linked to metabolic syndrome, for which the liver factors are indicators, is unclear. There is no current literature linking metabolic syndrome to Kdn metabolism. In a recent human study, Kdn was inversely correlated with BMI [45].

### 3.5 Sialic acid levels in mouse pup brain

We analyzed the Sia content in the pup brain collected at PN15, including 60 female and 61 male offspring. The total Sia (Neu5Ac, Neu5Gc, and Kdn) contents were expressed as ng/mg tissue in Fig. 4d–S-Table 5 and S-Table 6. There is no correlation between Sia concentrations in the brain tissue and total wet brain weight (p = 0.623). The total Sia level was around 543 ng/mg wet tissue in the control group (LF), of which Neu5Ac accounted for 495 ng/mg, Neu5Gc represented 44 ng/mg, and Kdn 3 ng/mg. As shown in Fig. 4d, no sex differences in mouse brain total Sia contents were found. The concentration of total Sia in our study is in line with the study of Wang et al. (1998), who reported that the content of total Sia was around 300 ng/mg in mouse brain at PN15 [26]. To note, the analytical methods used (Spectrophotometer vs HPLC-FLD in our study) might contribute to the slight differences in estimated Sia concentrations between our study and Wang et al.. Notably, the total Sia level in our study is much lower than reported by Malicdan et al. [25] who identified total Sia levels were 37,830 ng/mg (C57BL/6) in mouse brains. It has been proposed that total brain Sia level correlates with the brain maturation [26], hence the age of mice might affect the Sia concentration in the brain. Although Malicdan et al. studied mice aged between 0 and more than 40 weeks, the age of mice at which Sia concentration was analyzed was not specified. Gagiannis et al. [24] reported 13,561 ng/mg in brain cell membrane fractions of C57BL/6 mice. Importantly, these authors detected Sia via resorcinol coloring and UV absorption in pelletized brain membrane fraction, leading to values that cannot be directly compared with our results.

In accordance with previous studies in adult mice [25,46], Neu5Ac represented the major Sia while Neu5Gc was rare in pup brains (Fig. 4e), confirming a lower abundance of brain Neu5Gc content. Despite the application of the same analytical methods, the proportion of Nue5Gc in our study (∼8 %) is slightly higher than the data reported from previous studies, which showed that Neu5Gc only accounts for 0.8–3 % of total Sia content in mouse brain [46]. Again, as the mouse strain and age were not specified in that study, these parameters may explain differences in brain Sia content with our study. Besides, we detected a small amount of Kdn (<1 %) in mouse pup brain which, to the best of our knowledge, has not been reported before.

### 3.6 Maternal HIP and continued HF diet during lactation do not influence sia content in offspring brain

As there was no difference in Sia concentrations in mouse brains between female and male offspring (Supplemental Figure 4a), we combined females and males for statistical analysis. Moreover, as there was no difference between GDM and PDM mice, we combined GDM and PDM into a single HIP group.

**Fig. 6:**
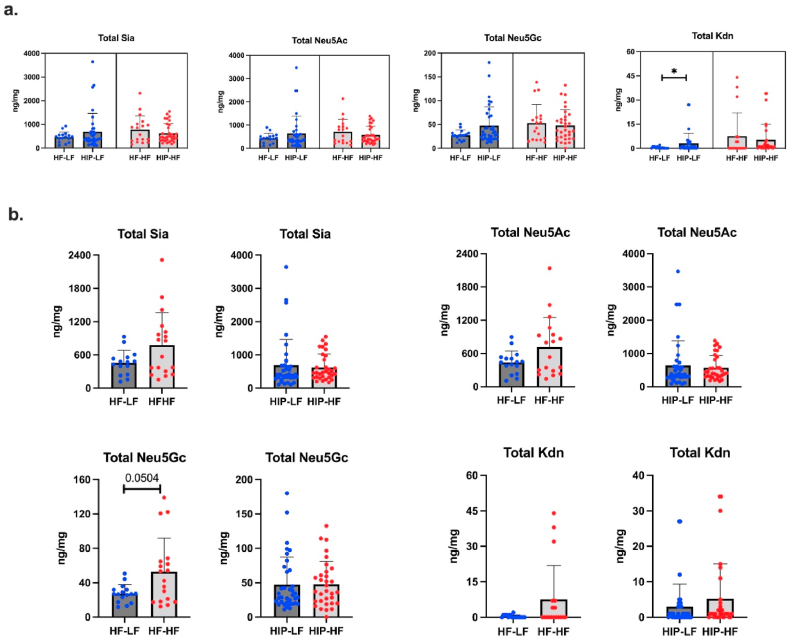
(a) The concentration of total brain Sia (Neu5Ac, Neu5Gc, Kdn) in LF (HF-LF, HIP-LF) and HF (HF–HF, HIP–HF) groups. (b) The concentration of total brain Sia (Neu5Ac, Neu5Gc, Kdn) in control (LF, HF) and HIP (LF, HF) groups. Blue points = low-fat diet during lactation, red points = high-fat diet during lactation. Kruskal Wallis followed by Dunn's multiple comparison test was used. *p < 0.05

Sia concentrations were slightly higher in HIP-LF than in HF-LF mice (Fig. 6a). However, when mice continued to receive an HF diet during lactation, Sia concentrations were somewhat lower in HIP–HF than in HF–HF mice (Fig. 6a). Although there were no significant differences between HIP and non-HIP pup brain Sia content, the trends in the offspring brain were in line with Sia concentrations in dam milk. Yet, these results suggest maternal HIP has no significant influence on Sia levels in offspring brains at PN15.

Next, we identified whether the continued HF diet during lactation influences Sia content in mouse brains (Fig. 6b). Pups from mothers who received HF diet during lactation showed higher brain Sia concentrations compared to offspring from mothers on an LF diet, although the difference was borderline statistically significant (*p* = 0.0504). LF diet switch during lactation did not affect the Sia level in the HIP-offspring brain. No significant differences were found in the Sia brain between offspring of LF-LF and HF-LF mothers (Supplemental Figure 4b).

Interestingly, we found that total and free Neu5Gc in mouse milk showed strong positive correlations with total Sia (Neu5Ac and Neu5Gc) concentrations in offspring brain (HF-LF and HF–HF group) (r > 0.5, *p* < 0.05) (Supplemental Table 8). This suggests that dietary Neu5Gc, while not a significant component in brain glycosylation, may influence Neu5Ac metabolism and incorporation into brain glycosylation in offspring. Whether Neu5Gc in mouse milk alters brain glycosylation in offspring, and whether Neu5Gc has biological significance in determining pups’ health outcomes remains to be established.

## 4 Conclusion

In conclusion, we quantified the milk oligosaccharide contents and milk and brain Sia levels in mouse dams and pups. PDM specifically affected sialylated oligosaccharides in mouse milk at PN15. Continued exposure of dams to HF during lactation influenced the content of the sialylated milk oligosaccharides compared to dams that received LF during lactation. Moreover, maternal HIP, regardless of severity and duration, significantly affected total and free Sia contents in mouse milk. HF diet during lactation (both in HF–HF and HIP–HF) significantly influenced the Neu5Gc level in mouse milk. Total and free Neu5Gc in mouse milk showed strong positive correlations with Sia (Neu5Ac and Neu5Gc) in the PN15 brain of offspring from HF-LF and HF–HF mouse dams. However, neither maternal HIP nor HF during lactation significantly affected Sia content in offspring's brain. These data may suggest that mouse milk Sia content exceeds the developmental needs of the pups and that even the lowest measured availability from milk in this study is sufficient to meet the requirements for brain development. The study has some weaknesses, primarily there is a limited amount of samples from the PDM group, as the aim of the original research was to generate GDM dams, and the PDM dams were an extra non-targeted group. This limited the number of dams in the PDM group and the statistical power. Whereas the original study performed a *pre-hoc* power calculation based on glucose tolerance, determining a group size of n = 9 to be sufficient, this power calculation was not explicitly performed toward milk composition. Secondly, the study collected milk only at PN15, at which timepoint the diabetic metabolic state was resolved in the majority of the GDM dams. Future studies should take milk samples at earlier stages of lactation before the metabolic state of the GDM dams is restored. Further studies should identify whether and how altered milk composition by HIP and HF diet impact later health outcomes in the offspring. Finally, it should be assessed how the significant differences in milk composition between mouse and human translates to the human situation. In principle, studying a human GDM population for milk composition should be feasible. The effects of milk composition on infant brain composition are much harder to study in a human context.

## Ethical considerations

Using samples from one mouse study for multiple research questions increases efficiency and helps reduce the use of experimental animals. Therefore, we used a suitable, pre-existing study, to investigate our research question. No new animals were used for our study, only materials and data from a previous study were employed.

## Funding

FL is supported by the China Scholarship Council (CSC) under Grant No. 201706350281.

## Data availability statement

Data will be provided on reasonable request.

## Additional information

No additional information is available for this paper.

## CRediT authorship contribution statement

**Fan Liu:** Writing – original draft, Visualization, Formal analysis, Data curation, Conceptualization. **Angela J.C. Tol:** Writing – review & editing, Validation, Investigation, Conceptualization. **Folkert Kuipers:** Writing – review & editing, Supervision. **Maaike H. Oosterveer:** Writing – review & editing, Validation, Supervision, Conceptualization. **Eline M. van der Beek:** Writing – review & editing, Supervision, Project administration, Conceptualization. **Sander S. van Leeuwen:** Writing – review & editing, Supervision, Methodology, Data curation.

## Declaration of competing interest

The authors declare the following financial interests/personal relationships which may be considered as potential competing interests: Fan Liu reports financial support was provided by China Scholarship Council.
