# Supplementary material for: Characterization of milk oligosaccharide and sialic acid content and their influence on brain sialic acid in a lean mouse model for gestational diabetes
Source: Heliyon. 2024 Jan 24;10(3):e24539. doi: 10.1016/j.heliyon.2024.e24539 (PMC10839809; doi:10.1016/j.heliyon.2024.e24539)
Supplement: Multimedia component 2 [file mmc2.pdf]

S-Figure 1.

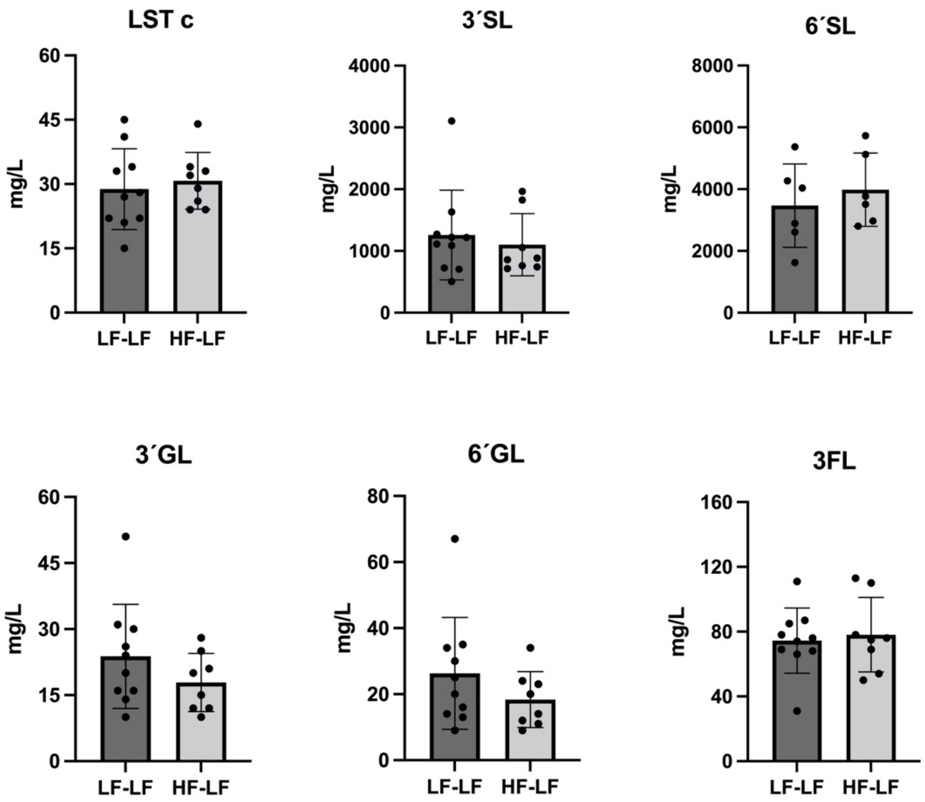

S-Figure 1. The concentration of oligosaccharides in LF-LF and HF-LF. Unpaired t test was used. **p*<0.05, ***p*<0.01, ****p*<0.001, *****p*<0.0001.

S-Figure 2.

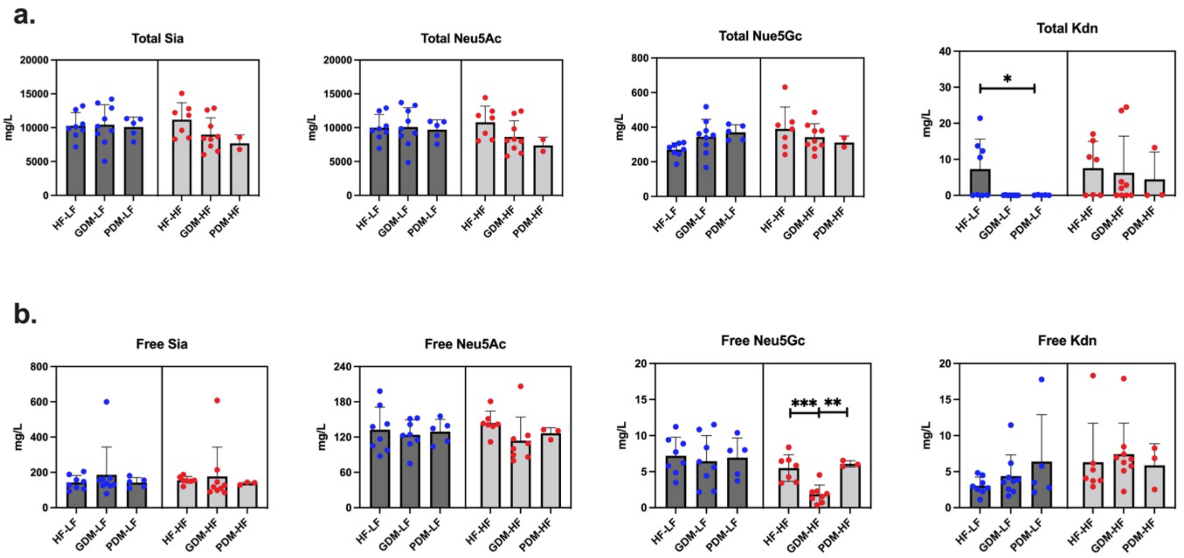

S-Figure 2. (a) The concentration of total milk Sia (Neu5Ac, Neu5Gc, Kdn) in LF (HF-LF, GDM-LF, PDM-LF) and HF (HF-HF, GDM-HF, PDM-HF) groups. (b) The concentration of free milk Sia (Neu5Ac, Neu5Gc, Kdn) in LF (HF-LF, GDM-LF, PDM-LF) and HF (HF-HF, GDM-HF, PDM-HF) groups. Blue points = low fat diet during lactation, red points = high fat diet during lactation. One-Way ANOVA followed by Tukey's multiple comparison test (Total Sia, Neu5Ac, Neu5Gc) or Kruskal Wallis followed by Dunn's multiple comparison test (Free Sia, Neu5Ac, Neu5Gc, Kdn, total Kdn) were used. **p* < 0.05, ***p* < 0.01, ****p* < 0.001.

S-Figure 3

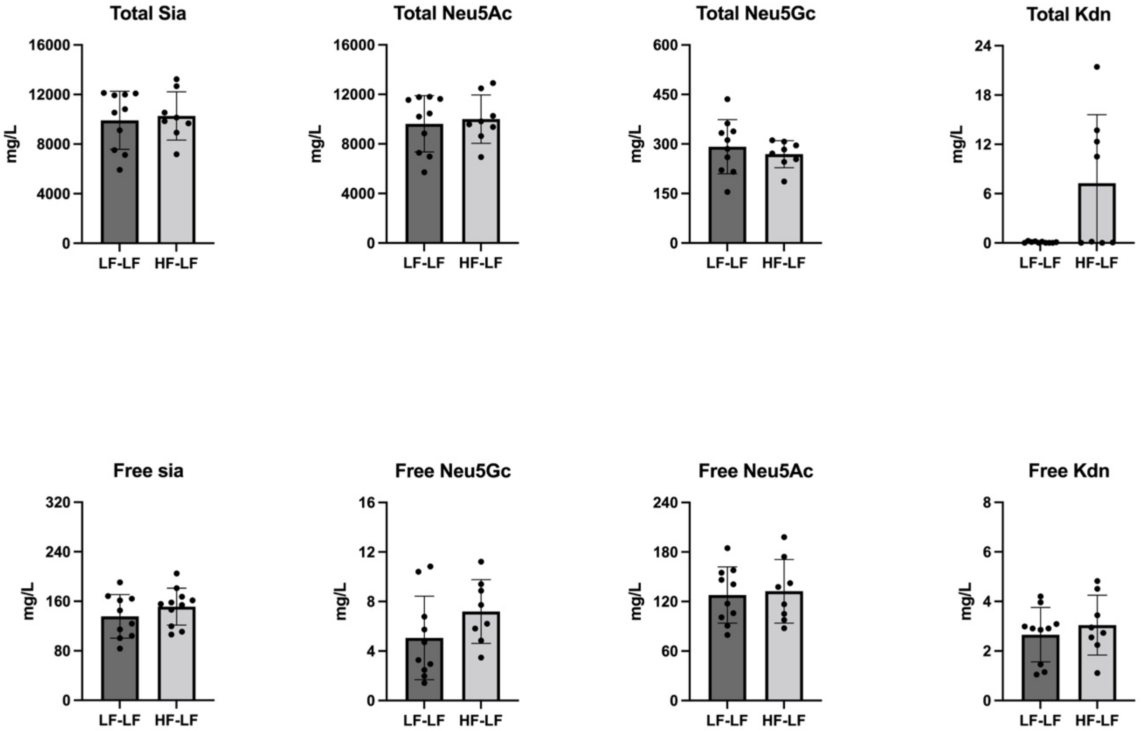

S-Figure 3. The concentration of total and free Sia (Neu5Ac, Neu5Gc, Kdn) in mouse milk between LF-LF and HF-LF groups. Unpaired t test (Total Sia, Neu5Ac, Neu5Gc) or Mann-Whitney test (Free Sia, Neu5Ac, Neu5Gc, Kdn, total Kdn) were used. **p*<0.05, ***p*<0.01, ****p*<0.001, *****p*<0.0001.

S-Figure 4

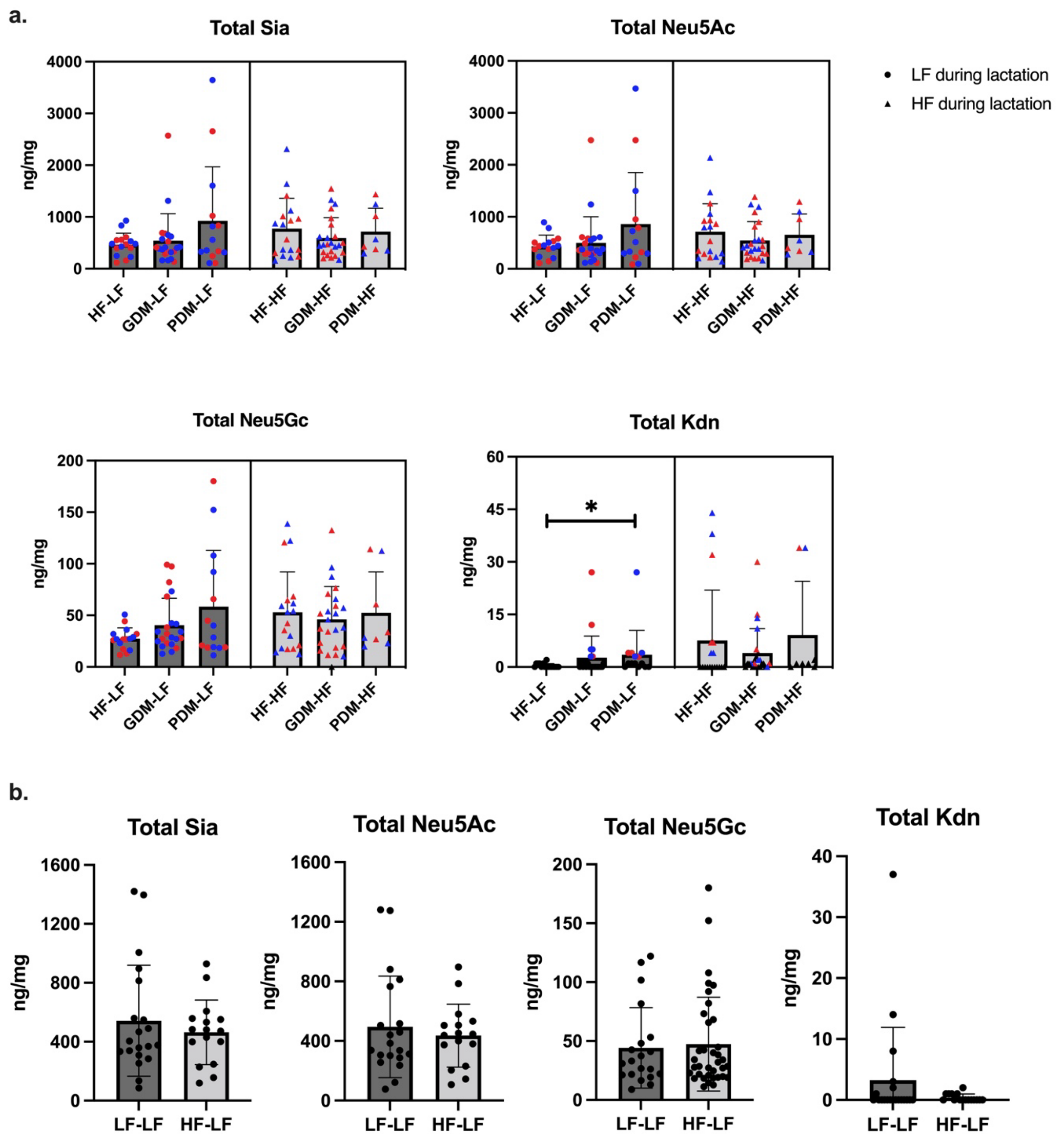

S-Figure 4. (a) The concentration of total brain Sia (Neu5Ac, Neu5Gc, Kdn) in LF (HF-LF, GDM-LF, PDM-LF) and HF (HF-HF, GDM-HF, PDM-HF) groups. (b) The concentration of total brain Sia in LF-LF and HF-LF groups. Blue points = male pups, red points = female pups. Round shape = low-fat diet during the lactation, triangle shape= high-fat diet during the lactation. Kruskal Wallis followed by Dunn's multiple comparison test were used (a), Mann-Whitney test was used (b). *p<0.05, **p<0.01, ***p<0.001, ****p<0.0001.
